# The District Dental Society of the 7th Judicial District of the State of N. Y.

**Published:** 1874-08

**Authors:** J. Edward Line


### Proceedings of Societies.

### THE DISTRICT DENTAL SOCIETY OF THE SEVENTH JUDICIAL DISTRICT OF THE STATE OF NEW YORK.

The above Society held its sixth annual session, at Rochester, N. Y., on the 2d and 3d of June, 1874.

The society was called to order by the President, Geo. W. Tripp, of Auburn.

Reports were reeeived from the Committee on Constitution and By-Laws, on essay and on business.

The first essay was on "Educatiou," by W. H. Atkinson, of New York. It was read by Dr. F. French, of Rochester.

An essay on the "Progressive and Retrograde Metamorphoses of Bones and Teeth," by S. P. Cutler, of Memphis, Tenn., was read by the Secretary.

An essay on "Rubber-Dam," by J. A. Chase, of Avon, was read by the author.

"The Dental Student," by Prof. J. Taft, of Cincinnati, O., was read by Dr. A. J. Kingsley, of Munda.

An essay on "The Proscription of the Schoolmen," by J. Edward Line, of Rochester, was read by the author.

Dr. L. D. Walter, of Rochester, presented a paper on the "Preparation of Cavities in Molar Teeth."

Dr. T. S. Hitchcock, of Seneca Falls, read an original paper with the title, "Fool Prints."

An essay entitled "A new Style of Pivot Teeth," by W. H. Shadoah, of Louisville, Ky., was read by Dr. H. S. Miller, of Rochester.

The gentlemen not members of the Society and who contributed essays were given a hearty vote of thanks.

A letter and circular were received from a committee of the American Dental Association, soliciting contributions to the Barnum testimonial fund.

Drs. J. Payne and L. D. Walter were appointed a committhe to bleed the brethren which they did to the amount of $22.50. This was placed in the hands of the Treasurer, with instructions to forward it to the proper parties.

The election resulted as follows:

President, H. S. Miller, Rochester;

Vice-President, B. R. McGregor, Rochester;

Secretary, J. Edward Line, Rochester;

Treasurer, T. E. Howard, Genessee;

District Censor for three years, T. S. Hitchcock, of Seneca Falls;

Delegates to State Society, D. F. Wilcox, B. F. Schuyler, C. Elmendorf.

The time of the meeting was so limited and the amount of miscellaneous business so great, that little attention could be given to the discussion of essays. These two facts will account partially for the meagerness of this sketch.

J. EDWARD LINE, Secretary.

